# National polydrug use patterns among people who misuse prescription opioids and people who use heroin. Results from the National Household Survey on Drug Use and Health

**DOI:** 10.1016/j.drugalcdep.2022.109553

**Published:** 2022-07-06

**Authors:** Georgiy V. Bobashev, Lauren K. Warren

**Affiliations:** RTI International, USA

**Keywords:** Polydrug patterns, Opioid misuse, National Survey on Drug Use and Health (NSDUH), Cluster analysis, Latent class analysis, Polysubstance

## Abstract

**Background:**

Polysubstance use among people who misuse opioids (PWMO) is highly prevalent, but understudied. We defined, estimated, and analyzed national polysubstance use patterns among PWMO using National Household Survey on Drug Use and Health data (2017–2019).

**Methods:**

We obtained estimates of past-month patterns of polydrug use using cluster analysis and latent class/profile analysis. We considered misuse of prescription opioids and use of heroin, cocaine (including crack), marijuana, alcohol, and “other” substances.

**Results:**

We identified a five-cluster solution for binary indicators of past-month use and a six-cluster solution for frequency of use. The largest binary cluster (37%) included misuse of prescription opioids and use of alcohol. The second-largest cluster (15%) included misuse of prescription opioids, alcohol, marijuana, and “other” substances. Among those who used heroin, 36% used methamphetamine.

In terms of frequency of use, the largest cluster among people who misuse opioid who used multiple substances (almost 40%) misused prescription pain relievers, alcohol, and marijuana infrequently. The second-largest cluster (23%) used marijuana almost daily and misused prescription pain relievers an average of 6.6 days. PWMO in a cluster of almost daily heroin use indicated use of methamphetamine, marijuana, and prescription opioids. Those who used methamphetamine, were using it more than 15 days a month.

**Conclusions:**

We have developed reference measures of polydrug patterns among US household population and estimated their demographic characteristics. We identified clusters of high-risk polydrug use. These findings have implications for the development of prevention and treatment solutions in the United States

## 1. Introduction

This paper describes national patterns of polysubstance use in National Survey on Drug Use and Health (NSDUH) data from 2017 through 2019 and relates them to representative demographics. Polysubstance use is a major health concern, especially when more than one substance is used simultaneously. The opioid epidemic is growing in the United States, and reports show that more than one drug accompanies overdoses (Dasgupta et al., 2018; Liu et al., 2021; McCabe et al., 2006; McCall Jones et al., 2017; National Institute on Drug Abuse, 2021; Penm et al., 2017). More worryingly, the involvement of multiple drugs in overdoses has increased. Over the last decade in the United States, overdoses involving both opioids and other drugs increased (Compton et al., 2021; Jalal et al., 2018; Liu et al., 2021). One of the main foci over the last decade was on combinations of opioids and stimulants and opioids and benzodiazepines (Kariisa et al., 2021; Liu et al., 2021). At the same time, alcohol and marijuana are the most prevalent substances, and a combination of them with other drugs could provide additional impairment and lead to injury, overdose, and death (Baggio, 2014; McCabe et al., 2006).

Polydrug patterns have previously been studied in local areas. For example, (Bobashev et al., 2018), five polydrug patterns were identified among polydrug users in Cleveland, OH. These patterns revealed that use varies from heavy (daily) use of multiple drugs together with heroin to occasional use of some drugs. In North Carolina, over 75% of overdose deaths involved more than one drug (Figgatt et al., 2021). Schneider et al. (Schneider et al., 2020, 2019) studied polysubstance use among people with opioid use disorder in Baltimore and West Virginia and emphasized the need to address the increased overdose risk among polysubstance users.

At the national level, (Agrawal et al., 2007) studied patterns of polydrug use from the National Epidemiological Survey on Alcohol and Related Conditions. Agrawal et al. (2007) and Compton et al. (2021) used NSDUH to estimate the number of drugs co-used in the lifetime. At the same time, patterns of polydrug use at the national level and especially among opioid users has not been systematically documented and summarized. One possible reason is that polydrug use is very complex to summarize because of its multiple dimensions: recency, frequency, concurrency, subpopulations/subclusters, outcomes (mortality, treatment, disorder, use), etc. Additionally, patterns can be defined with different analysis methods including prevalence reports of predefined combinations, cluster analyses, latent class, and latent profile analyses.

We address this challenge by focusing on concurrent past-month use, using a combination of 3 years of NSDUH data to obtain consistent estimates and employing cluster analysis together with latent class and profile analysis to show how different approaches uncover different sides of this complex problem. We further focus on a subsample of people who misuse opioids (PWMO) (i.e., individuals who use heroin or misuse prescription pain relievers).

## 2. Methods

### 2.1. Data: NSDUH 2017–2019

We considered analysis of recent NSDUH datasets, 2017–2019. NSDUH is an ongoing, annual cross-sectional survey designed to provide national estimates of substance use and mental health in the United States. The target population includes household residents from the 50 states (including shelters, rooming houses, and cluster homes; and civilians residing on military bases) and the District of Columbia. Participants are selected by representative multistage probability sample of respondents aged 12 years and older. The sample contains analysis weights that allow one to produce estimates at the national household level. More information about NSDUH datasets and methodology may be found in the survey’s technical documentation available online (SAMHSA, 2020). Survey items include over 3000 attributes covering demographics, health concerns, substance use, and mental health. The combined 2017–2019 public use datasets contained 168,725 interviews (roughly 55,000 per year). The survey covers many substances, including alcohol, marijuana, heroin, methamphetamine, cocaine (including crack), prescription psychotherapeutics (pain relievers, sedatives, tranquilizers, and stimulants), inhalants, and hallucinogens. Benzodiazepines were included in both prescription tranquilizers and prescription sedatives.

### 2.2. Measures

We defined past-month use of each substance based on the corresponding question in NSDUH. The variable indicating misuse of opioids was defined as either use of heroin in the past month or use of prescription pain relievers during the past month in a way a doctor did not recommend the individual to use them. The following seven substance categories were used in the analyses:

1. alcohol,
2. marijuana,
3. prescription pain relievers,
4. heroin,
5. cocaine (including crack),
6. methamphetamine, and
7. an “other” category, which included prescription sedatives, prescription stimulants, prescription tranquilizers, inhalants, and hallucinogens.

The “other” category includes the misuse of benzodiazepines in the prescription sedatives and prescription tranquilizers subcategories. Although benzodiazepines might significantly contribute to overdoses, it was not possible to separate benzodiazepines out of the “other” category when discussing past-month use. We did not include tobacco use in the definition of polysubstance use. Polysubstance use was defined as the use of two or more substances from the list of the seven substances above.

We considered two types of substance use measures: (1) binary (i.e., whether certain drugs or drug combinations were used in the past month in the entire population and in each of the clusters) and (2) count (i.e., reported numbers of days they used drugs in the past month). Although imperfect, number of days provides information about level of use (e.g., occasionally, daily). These two measures highlighted different aspects of drug use by targeting prevalence and frequency of use.

All substance use outcome and demographic variables used in our analyses had imputed versions available for use on the public use data files. These variables had been treated so that missing data were imputed using the predictive mean neighborhoods (PMN) or modified PMN methods. The imputation processes used in the NSDUH are described in detail in Section 2.3.3 of the NSDUH Methodological Summary and Definitions report (SAMHSA, 2020).

#### 2.2.1. Analysis

We considered two subpopulations for our analysis: all individuals with polydrug use (defined as use of two or more drugs in the past 30 days), and individuals who misused prescription pain relievers (in the past 30 days). For each subset, we performed clustering on two variations of the data: binary (indicator use or no use in the past 30 days) and number of days of use in the past 30 days. These two outcome measures highlighted different aspects of drug use by targeting prevalence and frequency of use.

When estimating the average number of days, the substance was used within each cluster, we considered two analyses: the average number of days the substance was used across all subjects in the cluster and the average number of days the substance was used only by those subjects who used the substance at all in the past month. The difference is minimal for more prevalent substances like alcohol and marijuana, but for low-prevalence drugs such as methamphetamine or cocaine, the difference could be large. If only a few people in the cluster use the drug daily, the cluster-level average number of days could be small, but among those who use, it is large.

To identify clusters and patterns of polydrug use and frequencies, we considered a cluster analysis and a latent class and profile analyses. Cluster analysis depends on the choice of distance measure (e.g., Euclidian, Manhattan) while Finite Mixture Models (FMM) depends on choice of distribution (e.g., multinomial, normal) and the shapes of the latent distributions (e.g., equal or varying variances). A popular k-means cluster analysis combines individuals into clusters in such a way that individuals belong to the cluster from which it has the shortest distance. Such clustering puts individuals straightforwardly into each of the well-defined clusters and it also allows a new person to be assigned to one of the existing clusters. The number of clusters was identified by computing the Jaccard measure of consistency and visually inspecting a “scree” plot of sum of squared error (SSE) for each number of clusters. In k-means analysis we used 100 random starting points and 1000 maximum iterations to obtain stable results. Sensitivity analysis was conducted using different seeds, and results were compared across runs to evaluate consistency.

We interpreted the patterns with respect to substance use and analyzed the demographic and precise substance usage characteristics within each cluster. We computed demographic tables for the clusters of each subset and calculated the means and standard errors using weighted analysis to reflect the proportion of the U.S. population. We used the final analysis weights in the public use files that reflect multistage sampling design. All analyses were conducted using R software.

As an alternative to cluster analysis, FMM estimates the distribution of distinct latent groups in the population and the probability that a person belongs to each of the groups. Each group is characterized by a specific distribution of outcome probabilities. For binary outcomes these groups are called classes and are estimated with latent class analysis (LCA). For continuous outcomes, these groups become latent profiles and are estimated with latent profile analysis (LPA). Thus, instead of calculating the distance from the cluster center as in k-means, FMM estimates the probabilities of belonging to a certain cluster. Although FMM estimates probabilities of belonging to a group, like in cluster analysis, an individual can be “hard assigned” to a group with which it has the highest probability.

FMM often has an advantage because it uses likelihood-based inferential formalism, such as goodness of fit and hypothesis testing, but interpretation becomes more distant because the classes/profiles are “latent” and not observed. Cluster analysis produces snapshot summaries of the data. For presentation purposes we used k-means as a primary analysis, but also conducted LCA/LPA to evaluate the sensitivity of the results to the choice of analysis method. The results of the LCA/LPA analyses are presented in Appendices A and B.

## 3. Results

### 3.1. Sample characteristics

Characteristics of the sample of PWMO and multiple substances from 2017 to 2019 are presented in Table 1. This table shows the sample distribution of common demographic characteristics and past-month use and frequency of use characteristics for single substances.

**Fig. 1.**
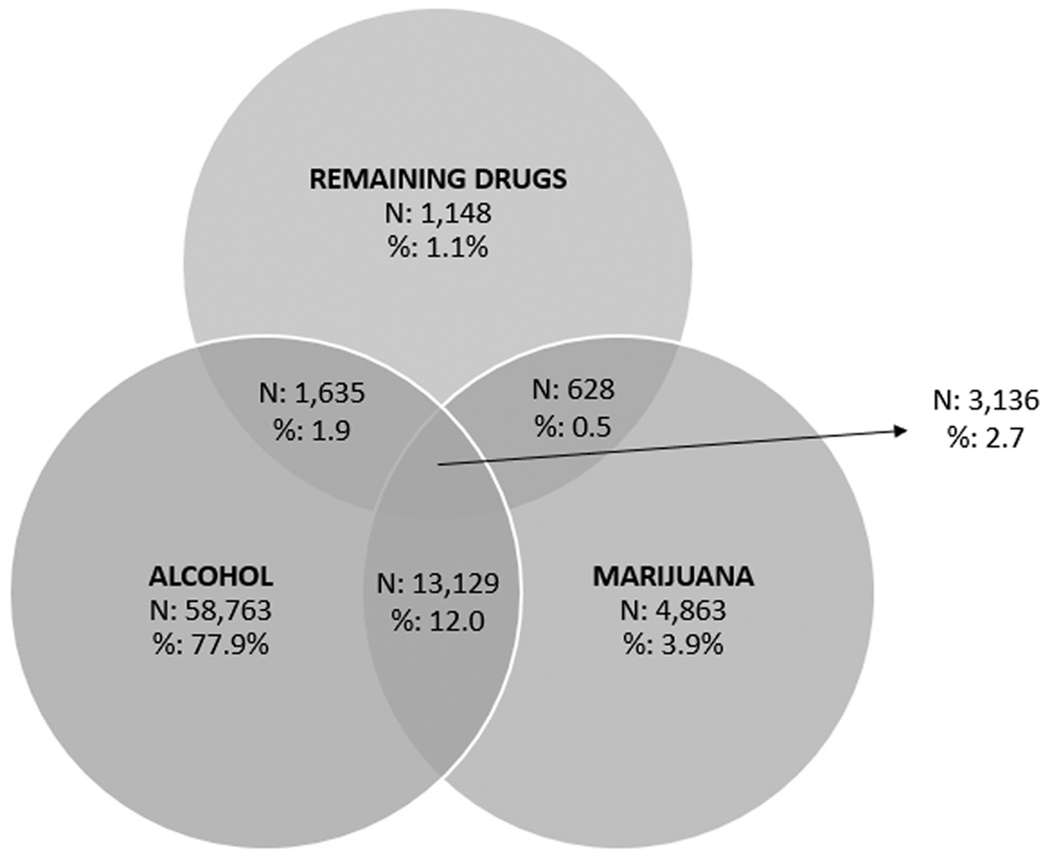
Past-Month Use of Alcohol, Marijuana, and Remaining Drugs Among All People Aged 12 or Older Who Used Illicit Drugs or Alcohol in the Past Month (N = 83,302), 2017–2019 NSDUH Note: The Ns in this figure are unweighted counts. The percentages in this figure are weighted to the national population.

In 2017–2019, polysubstance use in the previous 30 days was reported in 18,675 of the 168,725 respondents (9.3%). Fig. 1 shows that the most common substance pair was alcohol and marijuana, 12.0% (N = 13,129) among all individuals with past-month substance use and 69.5% of individuals with past-month use of multiple substances using the two substances exclusively and no others.

A total of 2195 respondents reported misuse of prescription pain relievers (including fentanyl patch) or use of heroin. Of these, 1789 respondents used more than one substance in the past month.

**Fig. 2.**
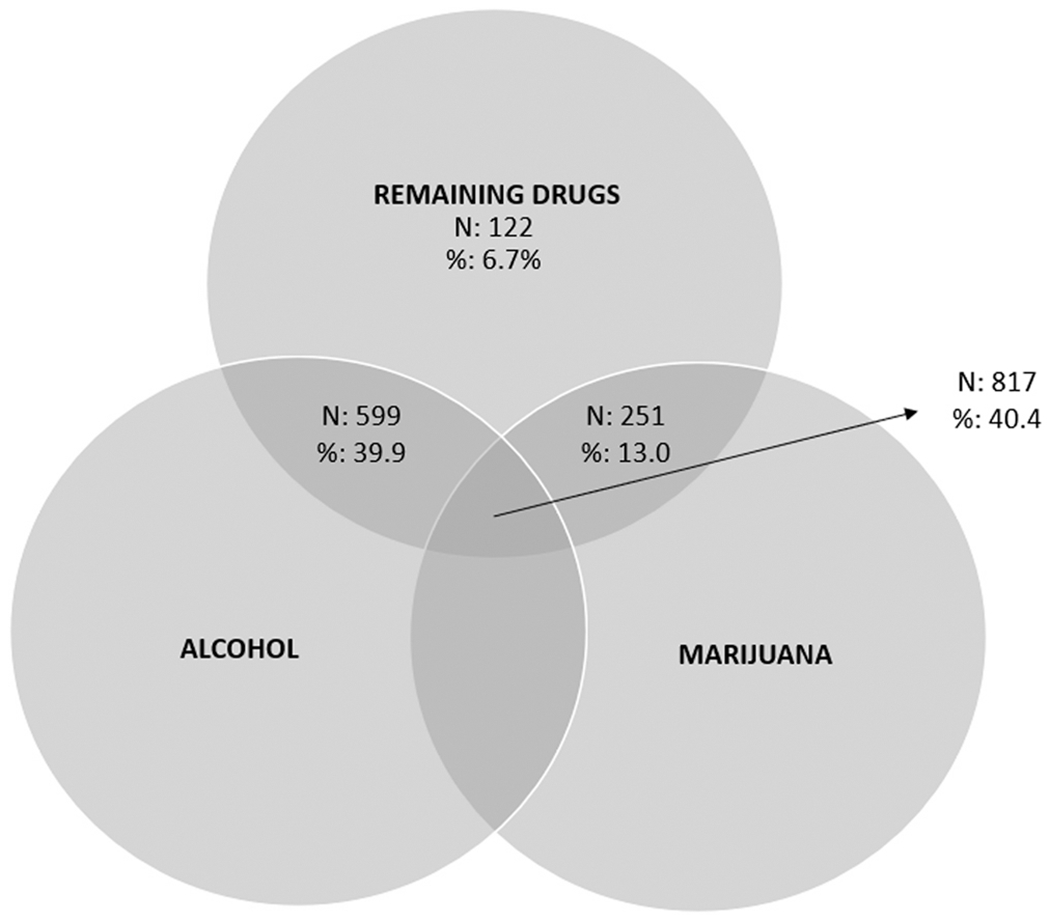
Past-Month Use of Alcohol, Marijuana, and Remaining Drugs Among PWMO and Multiple Substances Aged 12 or Older, 2017–2019 NSDUH Note: The Ns in this figure are unweighted counts. The percentages in this figure are weighted to the national population.

Fig. 2 shows the number and percentages of all PWMO and multiple substances who used combinations of alcohol, marijuana, and other drugs. Because multiple substances make up the “remaining drugs” category, including opioids, no PWMO and multiple substances fall into the alcohol only, marijuana only, or alcohol and marijuana sections of the Venn diagram. Fig. 2 shows that the most common combinations of substances among PWMO and multiple substances are (1) alcohol, marijuana, and remaining drugs (40.4%); and (2) alcohol and remaining drugs but not marijuana (39.9%). Respondents who fell into the remaining drugs only category had use of both prescription pain relievers and heroin or of prescription pain relievers or heroin and any another non-marijuana drug category. The breakdown of usage among those non-marijuana drug categories with opioids misuse are shown in the table on the left of the Venn diagram.

In further analysis, we focus on those 1789 PWMO who used multiple substance in the past month.

### 3.2. Binary usage patterns among PWMO and multiple substances

**Fig. 3.**
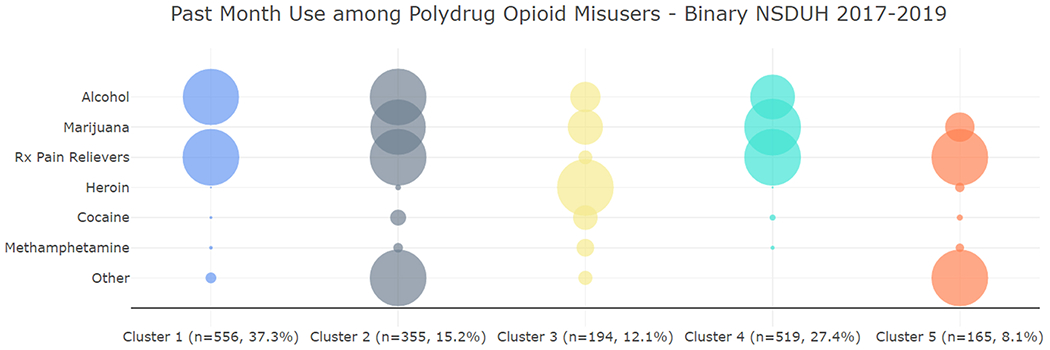
Patterns of Past-Month Substance Use Prevalence Among PWMO and Multiple Substances, 2017–2019 NSDUH.

The patterns of binary (i.e., past-month) polydrug use for those who misused prescription opioids or used heroin in the past month are presented in Fig. 3 and Table 2. The bubble plot indicates the probabilities that a person in the cluster used a drug in the last 30 days. The largest bubble corresponds to a prevalence of 1. A five-cluster solution for PWMO and multiple substances was chosen based on the stability analysis and cluster interpretation.

The majority of PWMO and multiple substances that made up the analysis sample misused prescription pain relievers (90.5%); however, the majority of people who used heroin (62.6%) did not report using prescription pain relievers. Fig. 3 and Table 2 show that Cluster 1 is the largest cluster, making up more than a third (37.3%) of the analysis population, and it is driven by alcohol use and high prescription pain reliever misuse (AP). Cluster 2 is characterized by use of prescription pain relievers, alcohol, and marijuana; relatively high proportions of cocaine (28.6%) and methamphetamine use (15.8%); and 100% of the cluster used “other drugs” (AMPO). All people in Cluster 3 used heroin (H), while all people in the other clusters misused prescription pain relievers but only a small fraction used heroin. Cluster 3 is also characterized by the highest proportions of cocaine (37.6%) and methamphetamine (36.1%) use among any of the clusters. The second largest cluster is Cluster 4, making up nearly a third (27.4%) of the analysis population and is characterized by 100% prescription pain reliever misuse and marijuana use, and relatively high alcohol use (78%) but no one in this cluster used “other” drugs and few people used heroin (1.2%) (AMP). Cluster 5 is the smallest cluster and is characterized by 100% prescription pain reliever use and 100% “other” drug use (PO). This cluster also has a relatively high prevalence of heroin (17.4%) and methamphetamine use (12.9%).

In terms of demographic characteristics, people in Cluster 3 (H) are less likely to be aged 12–17 and more likely to be male than the population average. People in Cluster 2 (AMPO) are more likely to be young adults (i.e., 18–34) and to be White than the population average. Cluster 5 (PO) is especially important because it is made up of 13.4% youths versus 4.8% youths in the total analysis population. Lower sample sizes for Clusters 2, 3, and 5 result in higher standard errors for weighted distributions of the demographic characteristics and therefore hinder the statistical comparisons of characteristics involving those groups.

### 3.3. Frequency usage patterns among PWMO and multiple substances

**Fig. 4.**
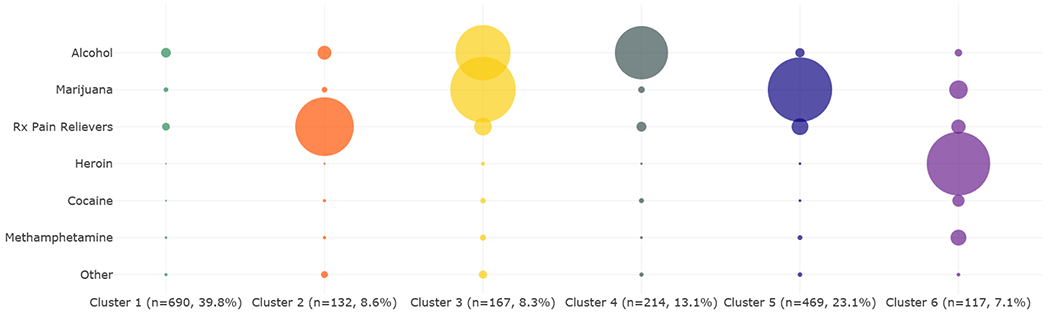
Patterns of Polydrug Use Among PWMO and Multiple Substances Based on the Number of Days Used Drugs in Past Month, 2017–2019 NSDUH.

Although binary substance use indicators partially describe patterns of substance use behaviors, another important component in understanding substance use patterns is the frequency of use for those drugs. For this analysis, past-month use is measured by the number of days someone used the substance in the past 30 days. We chose a six-cluster solution based on the k-means within-cluster SSE and stability assessment. Fig. 4 presents the bubble representation of the frequency of use patterns among PWMO and multiple substances. The largest circle corresponds to 30 days of use. The corresponding characteristics of the clusters are presented in Table 3.

Among the six clusters identified, Cluster 1 is the largest (39.8%) and is characterized by relatively low frequency of substance use. In this cluster, people misused prescription pain relievers an average of 2.8 days in the past month and used alcohol 3.8 days in the past month. The second largest cluster based on frequency of use is Cluster 5, which makes up 23.1% and is characterized by misuse of pain relievers more than twice as often as Cluster 1 and marijuana use nearly every day. Another important cluster is Cluster 6. Although small in size (7.1%), people in this cluster used heroin nearly every day in the past month. People in this cluster also used marijuana an average of 6.6 days in the past month and methamphetamine an average of 7.8 days in the past month.

In Cluster 6, which is characterized by heroin use, the dominant age group was 26–34 (42.9%), and over 70% of this cluster is male. This cluster is 78.7% White, compared to 68.7% of Whites that made up the population of PWMO and multiple substances. People in this cluster are more likely to be high school graduates (43.0%) and less likely to be college graduates (13.5%) than the population of PWMO and multiple substances overall (28.5% are high school graduates, and 20.1% are college graduates).

#### 3.3.1. Frequency of use limited to people who used substance

**Fig. 5.**
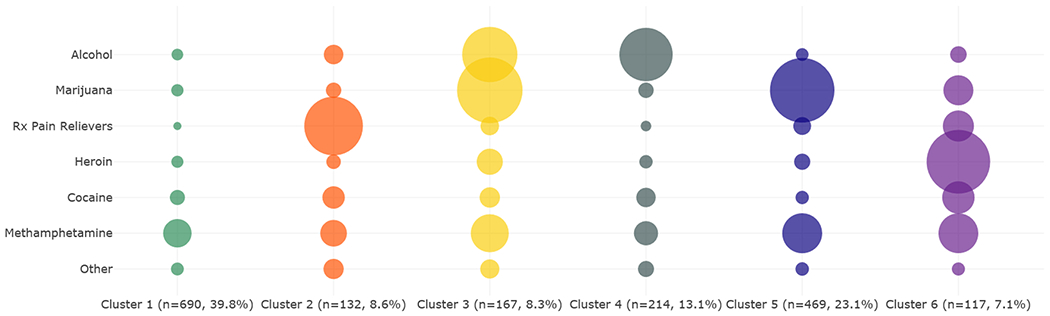
Patterns of Substance Use Among PWMO and Multiple Substances Based on Number of Days Used Substance in Past Month Conditioned on People Who Used the Substance in the Past Month, 2017–2019 NSDUH.

In Figs. 4 and 5, average days of use were measured over all respondents in the cluster. Those who did not use the drug contributed a value of zero. Fig. 5 presents patterns of use in polydrug opioid clusters where the average number of days was calculated only across individuals who actually used the drug (i.e., those who did not use the drug were excluded from the calculation and did not contribute zero values).

Comparing Figs. 4 to 5 reveals that for some drugs where the average frequency of use was low among people in a cluster, the frequency of use was relatively high among those who used the substance. This is indicated by the larger bubble sizes in Fig. 5 compared to Fig. 4. For example, the frequency of use for methamphetamine among all PWMO and multiple substances (Fig. 4) in Cluster 1 is 0.8 days, but the frequency of use among people who used methamphetamine in the past month in that cluster is 10.8 days (Fig. 5).

## 4. Discussion

This study presents definitions, estimation, and analysis of polysubstance use patterns from NSDUH. The specific focus was on the population who misused opioids in the past month. Past-month polysubstance use was prevalent in nearly 10% of the American noninstitutionalized household population. Marijuana and alcohol remain the most prominent polydrug combinations (over 85% of all people who used multiple substances used both alcohol and marijuana). Among PWMO, 81% in 2017–2019 used more than one drug in the past month.

In cluster analysis of polysubstance patterns defined by frequency of use, the largest cluster contained 39.8% of PWMO and multiple substances, and the majority of people in this cluster used the substances occasionally, just a few times a month (Fig. 4). In the cluster characterized by daily heroin use (12.1% of PWMO and multiple substances), use of other substances is relatively frequent with the most frequent being methamphetamine and cocaine, each of which was used by more than a third of the people in the cluster. Weighted proportions of each of the clusters were projected to the entire U.S. population and can be compared with national estimates for polydrug mortality; however, such analysis is beyond the scope of this study. The pattern results themselves are not sensitive to weighting because they indicate the most prominent combinations of substances used rather than representation of who is using these substances.

We considered two outcome definitions (binary and count) and two approaches (cluster analysis and FMM analysis). Different outcome definitions uncover the difference in patterns and highlight the variability of use intensity. A pattern of someone regularly using cocaine and occasionally heroin is substantially different from a pattern of daily use of heroin and occasional cocaine, although in binary assessment both patterns would be the same. Cluster analysis and FMM approaches, although different in nature of identifying groups, agree on the major patterns and highlight small groups of individuals who use multiple substances frequently.

Main classes/clusters that were identified could be interpreted as alcohol and prescription pain relievers (AP); prescription pain relievers and other (PO); alcohol, marijuana, and prescription pain relievers (AMP); alcohol, marijuana, prescription pain relievers, and other (AMPO); and mostly heroin (H). Those who used heroin also have high prevalence of methamphetamine and cocaine use. Although the approaches agree on the strongest signals, they treat smaller clusters differently. For example, CA did not distinguish a small cluster of 4% of individuals who used most of the substances being analyzed. At the same time, LCA rolled in three large groups—PO, AMP, and AMPO—into one class that could be interpreted as AMPO. We also considered a four-cluster CA solution to compare it with the four-class LCA solution (Appendix C). The four clusters were AP, AMP, AMPO, and H plus smaller probabilities of everything else. The LCA class that maps to the H cluster covers 13% of the sample and a class with high use of everything covered 4% of the sample.

Although LCA and CA use different approaches, their practical implementation here might not be as critically different. In fact, in LCA, one can assign an individual to a class based on the highest class probability and in CA, one can calculate a relative closeness to each cluster center, which will be equivalent to a probability belonging to a specific cluster. We considered a Davies-Bouldin measure of cluster separation, which estimates the average ratio between the sum of the size of each pair of clusters over the distance between the centers of these clusters. If all clusters are of the same width this measure becomes twice the measure of within to between cluster size. This measure is conservative because it selects pairs with the lowest distance between them. We replicated the analysis 100 times with different seeds and considered the mean value over the replications. The mean index was around 1.3 for four-cluster solution and 1.2 for five-cluster solution. Formal criteria of what constitutes an acceptably high degree of separation in LCA are not well defined, so we applied the DB measure to LCA maximum probability clusters to keep consistency in estimation. The separation was similar, although slightly worse with a value of 1.5.

With more people who use opioid in the United States than at any other time in history, it is important to recognize the heterogeneity of this population including demographics, regionality, and urbanicity. More specifically, identifying polysubstance use clusters among people who use opioid can be very useful in understanding the nature of the larger drug epidemic and how it can be addressed. The number of overdose deaths is growing, probably affected by the COVID-19 pandemic, so it is important to understand what polysubstance use patterns are represented among the U.S. household population. Our study developed an epidemiological reference basis for the national prevalence of drug combinations and identifies population subgroups with the most frequent polydrug use.

### 4.1. Limitations

A common limitation of a study based on survey data is reliance on self-reports. Additionally, the household-based study design does not consider the homeless population, where use of heroin and other drugs could be prevalent. Although NSDUH has obtained data from a large sample (>55,000 per year), only a small proportion (289 subjects in 2017–2019) reported use of heroin and multiple substances, and the results of cluster analysis specifically among people who used heroin are not presented here. This potential underreporting is a well-known issue.

It is possible that a different number of clusters, or even a different seed, would produce different clusters. We therefore conducted sensitivity analysis using different seeds and bootstrap replications. Four-cluster solutions were quite stable, while a five-cluster solution separates a PO cluster, but in some cases produced a small cluster that contains the use of almost everything. In the interpretation of the analysis, we selected a five-cluster solution with PO as a separate cluster because it had a better separation coefficient but also mentioned a four-cluster alternative solutions.

The study did not consider the use of benzodiazepines as a separate drug class, although they might significantly contribute to overdoses; thus their misuse, especially with opioids, is of high interest. This limitation is because of the lack of specific coding for benzodiazepines in the questionnaire. Nevertheless, benzodiazepines were included in “other” substances as sedatives and tranquilizers; thus, co-use of “other” substances is inclusive of benzodiazepines but might require a separate analysis.

Despite the limitations, our findings have implications for the development of prevention and treatment solutions in the United States. From a prevention perspective, understanding multiple drug use highlights the interplay between recreational and problematic use of multiple substances with opioids. From a treatment perspective, polydrug use poses a bigger challenge than just treatment for opioid dependence. The presented methodology is generic and allows one to uncover polydrug patterns in a broad range of datasets.

## Figures and Tables

**Table 1:** Characteristics of the 2017–2019 Public Use NSDUH Sample Among PWMO and Multiple Substances, N = 1789.

Demographic/Substance Use Characteristic	PWMO and Multiple Substances Weighted Percentage or Average (Standard Error)
**Age**	
12–17	4.8 (0.43)
18–25	19.4 (1.01)
26–34	25.4 (1.38)
35–49	25.8 (1.35)
50 +	24.5 (1.89)
**Gender**	
Male	56.5 (1.76)
Female	43.5 (1.76)
**Race**	
White	68.7 (1.66)
Black	11.8 (1.37)
Native American/Alaska Native	0.6 (0.11)
Native Hawaiian/Other Pacific Islander	0.4 (0.15)
Asian	1.7 (0.62)
Two or More Races	2.6 (0.41)
Hispanic	14.3 (1.25)
**Education (18 +)**	
Less than high school	11.1 (1.03)
High school graduate	28.5 (1.60)
Some college	35.4 (1.73)
College graduate	20.1 (1.49)
**Past-Month Substance Use**	
Past-month alcohol use	80.3 (1.31)
Past-month marijuana use	53.3 (2.04)
Past-month Rx pain reliever misuse	90.5 (1.24)
Past-month heroin use	15.8 (1.46)
Past-month cocaine use	13.1 (1.09)
Past-month methamphetamine use	10.7 (1.13)
Past-month other use	30.7 (1.83)
**Past-Month Use Frequency (Average Days per Month)**	
Days used alcohol in past month	7.9 (0.32)
Days used marijuana in past month	9.6 (0.47)
Days misused Rx pain relievers in past month	6.3 (0.29)
Days used heroin in past month	2.3 (0.23)
Days used cocaine in past month	1.0 (0.12)
Days used methamphetamine in past month	1.5 (0.22)

**Table 2:** Characteristics of the 2017–2019 Public Use NSDUH Sample Among PWMO and Multiple Substances, by Cluster Assignment, N = 1789.

Demographics/Substance Use	Cluster 1 Weighted Percentage: 37.3%	Cluster 2 Weighted Percentage: 15.2%	Cluster 3 Weighted Percentage: 12.1%	Cluster 4 Weighted Percentage: 27.4%	Cluster 5 Weighted Percentage: 8.1%
**Age (SE)**					
12–17	2.9 (0.59)	9.0 (1.33)	0.9 (0.44)	4.4 (0.84)	13.4 (2.77)
18–25	12.3 (1.21)	35.9 (3.32)	12.6 (2.89)	23.5 (1.65)	17.6 (3.01)
26–34	20.8 (1.99)	34.5 (3.54)	30.7 (5.42)	23.1 (2.46)	29.8 (5.31)
35–49	27.4 (2.16)	15.8 (2.58)	25.3 (3.75)	31.3 (2.87)	19.1 (3.04)
50 +	36.6 (2.47)	4.7 (2.92)	30.5 (6.43)	17.7 (3.19)	20.0 (6.66)
**Sex (SE)**					
Male	51.1 (2.97)	62.7 (3.59)	70.8 (5.14)	56.2 (2.69)	49.3 (6.02)
Female	48.9 (2.97)	37.3 (3.59)	29.2 (5.14)	43.8 (2.69)	50.7 (6.02)
**Race (SE)**					
White	66.1 (2.35)	76.5 (2.97)	72.2 (6.35)	65.8 (2.97)	70.7 (5.08)
Black	11.7 (1.97)	8.7 (2.26)	15.2 (6.85)	13.9 (2.06)	5.7 (1.74)
Native American/Alaska Native	0.5 (0.17)	1.3 (0.60)	0.3 (0.20)	0.6 (0.21)	0.3 (0.19)
Native Hawaiian/Other Pacific Islander	0.5 (0.31)	0.0 (*)	0.0 (*)	0.5 (0.44)	0.7 (0.57)
Asian	3.4 (1.56)	1.0 (0.55)	0.4 (0.37)	0.8 (0.38)	0.0 (*)
Mixed	1.6 (0.61)	3.7 (0.96)	3.1 (1.79)	3.1 (0.91)	3.0 (0.99)
Hispanic	16.3 (1.98)	8.9 (1.77)	8.9 (3.11)	15.3 (2.82)	19.6 (4.96)
**Education for 18 + (SE)**					
Less than high school	10.0 (1.76)	12.1 (2.85)	13.2 (2.97)	10.0 (1.39)	15.4 (3.58)
High school graduate	23.6 (3.10)	29.6 (3.35)	38.9 (4.69)	29.8 (3.02)	29.0 (4.76)
Some college	36.8 (2.89)	37.3 (3.42)	28.8 (3.92)	38.0 (2.90)	26.5 (6.27)
College graduate	26.8 (2.97)	11.9 (2.76)	18.2 (5.11)	17.8 (1.94)	15.7 (5.48)
**Past-Month Substance Use**					
Past-month alcohol use	99.1 (0.43)	100.0 (*)	56.0 (6.12)	78.0 (2.97)	0.0 (*)
Past-month marijuana use	0.0 (*)	97.7 (0.87)	56.2 (4.58)	100.0 (*)	54.2 (5.30)
Past-month Rx pain reliever misuse	100.0 (*)	100.0 (*)	21.3 (3.97)	100.0 (*)	100.0 (*)
Past-month heroin use	1.8 (0.67)	9.0 (2.10)	100.0 (*)	1.2 (0.25)	17.4 (4.15)
Past-month cocaine use	3.8 (1.22)	28.6 (3.14)	37.6 (4.66)	8.7 (1.73)	5.6 (1.94)
Past-month methamphetamine use	3.3 (0.79)	15.8 (2.76)	36.1 (6.35)	6.1 (1.41)	12.9 (3.76)
Past-month other drug use	13.1 (1.83)	100.0 (*)	21.2 (4.10)	0.0 (*)	100.0 (*)

(*) Standard errors are not included because of insufficient data in this cell.

**Table 3:** Demographics of PWMO and Multiple Substances by Clusters Based on Frequency of Use in Past Month, 2017–2019 NSDUH.

Demographics/Substance Use	Cluster 1 Weighted Percentage: 39.8%	Cluster 2 Weighted Percentage: 8.6%	Cluster 3 Weighted Percentage: 8.3%	Cluster 4 Weighted Percentage: 13.1%	Cluster 5 Weighted Percentage: 23.1%	Cluster 6 Weighted Percentage: 7.1%
**Age (SE)**						
12–17	7.1 (0.86)	1.8 (0.83)	2.3 (0.68)	1.3 (0.67)	6.4 (0.86)	0.4 (0.40)
18–25	17.0 (1.33)	11.4 (2.56)	30.3 (4.65)	10.2 (1.59)	28.6 (2.80)	17.6 (3.88)
26–34	20.3 (2.00)	30.4 (4.87)	33.4 (5.20)	23.6 (3.55)	25.2 (2.20)	42.9 (6.39)
35–49	25.1 (2.10)	28.2 (5.36)	29.4 (5.51)	30.9 (4.60)	23.0 (2.38)	22.7 (5.41)
50 +	30.6 (3.31)	28.3 (7.19)	4.6 (2.85)	34.0 (4.76)	16.8 (3.42)	16.4 (5.74)
**Sex (SE)**						
Male	48.7 (2.34)	53.1 (6.81)	63.3 (5.16)	54.9 (4.43)	65.2 (3.39)	71.0 (5.19)
Female	51.3 (2.34)	46.9 (6.81)	36.7 (5.16)	45.1 (4.43)	34.8 (3.39)	29.0 (5.19)
**Race (SE)**						
White	61.3 (2.66)	72.4 (5.30)	75.7 (4.56)	75.6 (3.97)	70.6 (3.06)	78.7 (4.74)
Black	12.9 (2.42)	6.4 (2.77)	12.9 (3.89)	14.4 (3.09)	12.0 (1.71)	5.4 (2.67)
Native American/Alaska Native	0.8 (0.23)	0.7 (0.43)	0.9 (0.49)	0.3 (0.17)	0.4 (0.22)	0.0 (*)
Native Hawaiian/Other Pacific Islander	0.8 (0.37)	0.0 (*)	0.0 (*)	0.3 (0.31)	0.1 (0.06)	0.0 (*)
Asian	3.2 (1.48)	1.9 (1.45)	0.0 (*)	0.6 (0.38)	0.8 (0.47)	0.0 (*)
Mixed	2.3 (0.60)	3.8 (2.10)	2.9 (0.96)	0.6 (0.33)	4.3 (1.06)	0.9 (0.55)
Hispanic	18.8 (2.15)	14.8 (4.31)	7.6 (2.73)	8.2 (2.35)	11.9 (2.40)	14.9 (4.56)
**Education for 18 + (SE)**						
Less than high school	11.3 (1.69)	15.9 (4.24)	11.2 (2.46)	5.5 (1.80)	11.9 (2.19)	12.3 (3.69)
High school graduate	21.9 (2.51)	26.6 (5.64)	28.1 (4.17)	34.2 (5.35)	33.0 (3.23)	43.0 (5.58)
Some college	34.9 (3.02)	35.2 (6.08)	42.2 (4.95)	34.5 (4.52)	36.0 (3.68)	30.7 (5.74)
College graduate	24.9 (2.75)	20.6 (6.81)	16.2 (4.00)	24.5 (4.82)	12.7 (2.63)	13.5 (5.29)
**Past-Month Substance Use**						
Past-month alcohol use	84.3 (1.82)	74.4 (5.47)	100.0 (*)	100.0 (*)	68.3 (3.15)	44.2 (7.05)
Past-month marijuana use	27.6 (2.51)	28.9 (6.42)	100.0 (*)	32.9 (4.53)	100.0 (*)	58.7 (6.09)
Past-month Rx pain reliever misuse	92.4 (2.19)	100.0 (*)	95.7 (2.81)	93.7 (1.90)	94.8 (1.13)	42.5 (5.63)
Past-month heroin use	9.9 (2.35)	9.6 (3.38)	8.2 (3.10)	8.5 (2.44)	9.3 (1.80)	100.0 (*)
Past-month cocaine use	7.4 (1.66)	9.1 (3.39)	20.2 (4.35)	18.5 (3.86)	14.0 (2.25)	29.3 (5.80)
Past-month methamphetamine use	7.1 (1.61)	3.7 (1.45)	11.0 (2.95)	5.4 (1.85)	10.2 (1.98)	49.9 (6.19)
Past-month other drug use	20.3 (2.13)	45.3 (7.02)	49.0 (6.30)	21.5 (3.10)	41.2 (3.67)	32.3 (6.36)
**Past-Month Use Frequency (Average Days per Month)**						
Alcohol use	3.8 (0.19)	6.5 (1.63)	23.1 (0.58)	22.0 (0.52)	3.3 (0.23)	3.4 (0.84)
Marijuana use	1.2 (0.14)	1.9 (0.57)	26.8 (0.35)	1.7 (0.32)	26.1 (0.33)	6.6 (0.86)
Rx pain reliever misuse	2.8 (0.18)	23.7 (0.69)	6.7 (0.78)	3.9 (0.35)	7.4 (0.53)	5.6 (0.91)
Heroin use	0.4 (0.10)	0.4 (0.14)	1.2 (0.60)	0.5 (0.21)	0.7 (0.18)	25.7 (0.71)
Cocaine use	0.4 (0.16)	0.6 (0.23)	1.3 (0.28)	1.8 (0.53)	0.6 (0.13)	4.0 (0.96)
Methamphetamine use	0.8 (0.24)	0.3 (0.12)	1.7 (0.54)	0.6 (0.34)	1.8 (0.41)	7.8 (1.63)
**Past-Month Use Frequency Conditioned on People Who Used the Substance in the Past Month (Average Days per Month)**						
Alcohol use	4.5 (0.21)	8.7 (2.01)	23.1 (0.58)	22.0 (0.52)	4.8 (0.25)	7.7 (1.09)
Marijuana use	4.5 (0.30)	6.6 (1.19)	26.8 (0.35)	5.3 (0.57)	26.1 (0.33)	11.2 (1.19)
Rx pain reliever misuse	3.0 (0.17)	23.7 (0.69)	7.0 (0.79)	4.2 (0.37)	7.8 (0.55)	13.2 (1.24)
Heroin use	3.9 (0.64)	4.6 (1.08)	14.2 (2.85)	6.2 (1.13)	7.1 (1.04)	25.7 (0.71)
Cocaine use	6.1 (1.53)	6.4 (1.28)	6.4 (0.94)	9.6 (1.37)	4.1 (0.68)	13.6 (1.71)
Methamphetamine use	10.8 (2.63)	7.9 (1.80)	15.8 (2.65)	10.7 (4.00)	17.6 (1.93)	15.7 (2.35)

(*) Standard errors are not included because of insufficient data in this cell.

**Fig. A.1.**
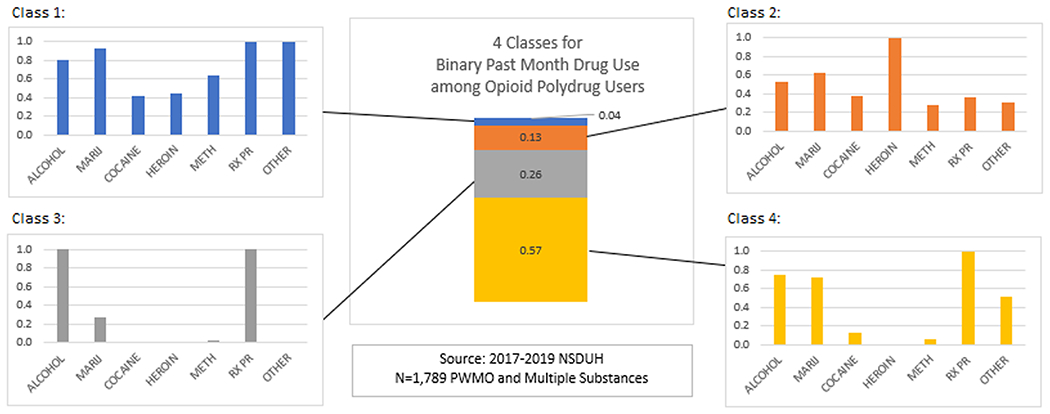
Class-Conditional Response Probabilities Output From the Latent Class Analysis Model for the Seven Substance Categories, 2017–2019 NSDUH. Note: MARI*J* = Marijuana; METH = Methamphetamine; RX PR = Prescription Pain Relievers.

**Fig. A.2.**
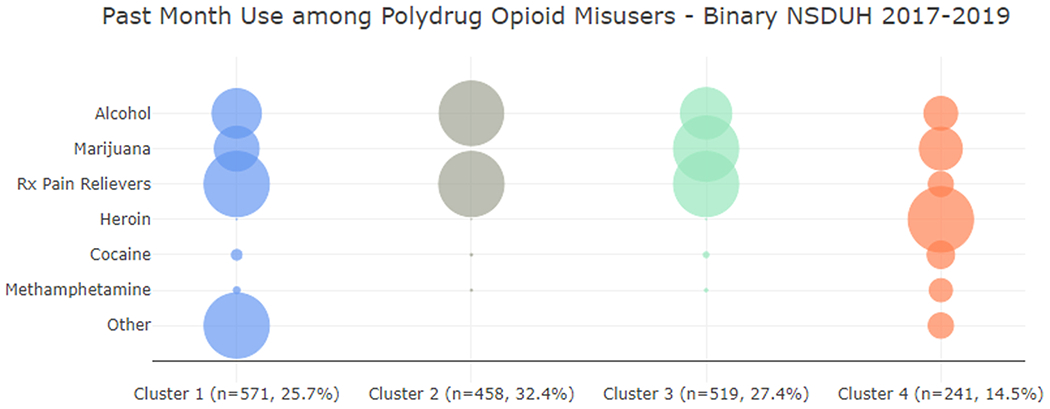
Patterns of Past-Month Substance Use Prevalence Among PWMO and Multiple Substances; Results of Cluster Analyses with Four-Cluster Solution for the Seven Substance Categories, 2017–2019 NSDUH.

**Fig. B.1.**
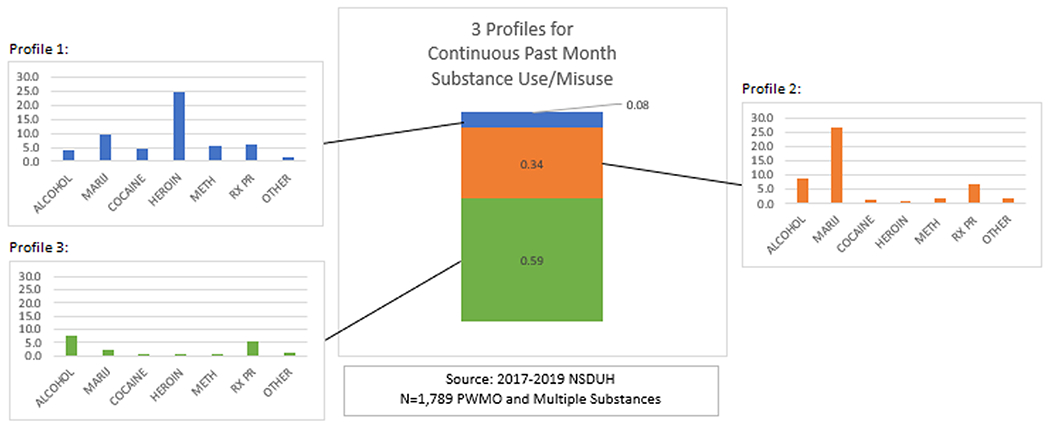
Model-Estimated Number of Days Used/Misused Substances in Past Month for Each Profile from the Latent Profile Analysis, 2017–2019 NSDUH. Note: MARI*J* = Marijuana; METH = Methamphetamine; RX PR = Prescription Pain Relievers.

## Appendix A. Latent class analysis results among PWMO and multiple substances

The best performing latent class analysis model based on the BIC was the 4-class solution. Figure A.1 shows the estimated class-conditional response probabilities for each of the four estimated classes. These are represented in the bar charts associated with each latent class. The size of each class is based on the mixing proportions output from the LCA model. Because the population for this analysis is all PWMO and multiple substances, every person included in the analysis used either prescription pain relievers or heroin (or both) and used more than one substance.

The smallest class, Class 1, makes up only 4% (standard error 1%) of the PWMO and multiple substances; however, this class has the highest number of substances used in the past month (an average of 5.83 substances). This class has relatively high use of heroin (44%) based on the class-conditional response probabilities and has the highest overall rates of other substances. This class has 100% use of prescription pain relievers and 100% use of “other” drugs. Other drugs include tranquilizers and sedatives, both of which include misuse of prescription benzodiazepines, if applicable. The percentage of alcohol use in this class is 81%, marijuana use is 92%, and methamphetamine is 64%.

Class 2 makes up 13% (standard error 1%) of the analysis population and is characterized by heroin use at 100%. In this class, the percentage of use of the other substances are alcohol use at 53%, marijuana use at 62%, cocaine use at 38%, methamphetamine use at 27%, pain relievers at 36%, and “other” drugs at 30%. This is another important class of people with polydrug use because of the high heroin use and relatively high use of other substances.

The other two classes—and the majority of the population of PWMO and multiple substances—used pain relievers (not heroin). Class 3 makes up 26% of the population (standard error 3%) and is driven by alcohol use and prescription pain reliever misuse and low use of other drugs. Class 4 is the largest cluster and involves the use of additional substances with marijuana use at 72%, alcohol use at 75%, cocaine use at 13%, and “other” drugs at 51% Figs. A.1 and A.2.

Based on predicted class assignment, Class 1 has the highest number of substances (among the seven substance use categories described in the Methods section) used in the past month with 5.83 substances. The smallest number of substances used is in Class 3 with an average of 2.03 substances used/misused (mainly alcohol and prescription pain relievers). By far the largest class, Class 4, is characterized by over three substances used/misused in the past month.

We conducted a separate four-cluster analysis, to compare with four-class LCA. The results are presented in Fig. A.2.

## Appendix B. Latent profile analysis

For analysis of the continuous measures of past-month number of days used substances, a latent profile model was applied using tidyLPA in R software. A final model with three profiles was deemed the best performing model based on AIC, BIC, and Entropy statistics. Assumptions of zero covariance and equal variances were set to avoid model convergence and estimation warnings. The models were run using scaled and unscaled data, and the results were the same.

The smallest profile in size makes up less than 10% of the cluster of PWMO and multiple substances but is characterized by a high number of days using heroin (nearly 25 days in the past month). Along with heroin, this profile is characterized by usage of other substances such as marijuana on 10 days in the past month and methamphetamine and prescription pain relievers on nearly 6 days each. Profile 3 is almost 60% of the PWMO and multiple substances and is characterized by use of alcohol and misuse of prescription pain relievers one to two times a week (7.5 days in past month for alcohol and 5.6 days for prescription pain relievers in past month). The remaining third of the PWMO and who misused multiple substances is characterized by alcohol use (8.5 days) and pain reliever misuse (6.9 days) along with nearly daily use of marijuana (26.7 days) Fig. B.1.

## Abbreviations:

NSDUH: National Survey on Drug Use and Health)
PWMO: people who misuse opioids
i.e.: individuals who use heroin or misuse prescription pain relievers
SSE: sum of squared error
LPA: latent profile analysis
LCA: latent class analysis
FMM: finite mixture models
